# Living Kidney Donation Practices in Europe: A Survey of DESCaRTES and EKITA Transplantation Working Groups

**DOI:** 10.3389/ti.2025.14802

**Published:** 2025-07-15

**Authors:** Marco van Londen, François Gaillard, Gianluigi Zaza, Gabriel C. Oniscu, Ilaria Gandolfini, Lucrezia Furian, Jelena Stojanovic, David Cucchiari, Luuk B. Hilbrands, Geir Mjøen, Christophe Mariat

**Affiliations:** ^1^ Department of Internal Medicine, Division of Nephrology, University Medical Center Groningen, Groningen, Netherlands; ^2^ Édouard Herriot Hospital, Hospices Civils de Lyon (HCL), Lyon, France; ^3^ Renal, Dialysis and Transplant Unit, Department of Pharmacy and Health and Nutrition Sciences, University of Calabria, Rende, Italy; ^4^ Edinburgh Transplant Centre, Royal Infirmary of Edinburgh, Edinburgh, United Kingdom; ^5^ Division of Transplantation, Department of Clinical Science, Intervention and Technology (CLINTEC), Karolinska Institutet, Stockholm, Sweden; ^6^ Nephrology Unit, University Hospital of Parma, Department of Medicine and Surgery, University of Parma, Parma, Italy; ^7^ Kidney and Pancreas Transplantation Unit, Department of Surgical Gastroenterological and Oncological Sciences, University Hospital of Padua, Padua, Italy; ^8^ Renal Unit, Great Ormond Street Hospital for Children NHS Foundation Trust, London, United Kingdom; ^9^ Department of Nephrology and Kidney, Transplantation Hospital Clinic, Barcelona, Spain; ^10^ Department of Nephrology, Radboud University Medical Center, Nijmegen, Netherlands; ^11^ Department of Nephrology, Lovisenberg Hospital, Oslo, Norway; ^12^ Department of Nephrology, Dialysis and Renal Transplantation, Centre Hospitalier Universitaire de Saint Etienne, Université Jean MONNET, Saint Etienne, France

**Keywords:** kidney function, living kidney donation, donor screening, risk assessment, donor follow-up

## Abstract

Thorough evaluation of potential kidney donors ensures safety and graft quality, but European data on donor practices are lacking. An online survey was conducted to assess European practices regarding kidney function, risk assessment and follow-up. 56% of respondents (125 practitioners, 16 countries, ∼3700 donations annually) use eGFR_CKD-EPI_, 34% use creatinine clearance and 70% use measured GFR. Sixty-three percent have no upper age limits, 91% exclude candidates with hypertension with end-organ damage, and 78% candidates on ≥2 antihypertensives. BMI cut-offs of 30 (39%) and 35 kg/m^2^ (42%) are common. Candidates are excluded for an HbA1c ≥ 53 mmol/mol (46%), glucose ≥7 (57%) or ≥11.1 mmol/L after glucose-tolerance test (59%). ApoL1-testing is not routine in 73%, and 38% perform a kidney biopsy if albuminuria/hematuria is present. Spot and 24-hour urine albumin is assessed in 38%. Hematuria is accepted when urological evaluation (15%), kidney biopsy (16%), or both (57%) are normal. Low-risk stones often do not preclude donation. Written informed consent is obtained by 95% of centers, with 65% asking consent for data. Lifetime follow-up is offered by 83%. This first study on evaluation and follow-up practices of donors in Europe shows variation between centers, suggesting a need for harmonization of donor practices.

## Graphical Abstract

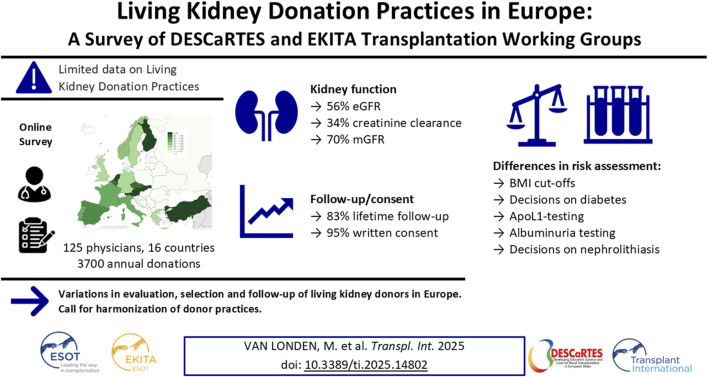

## Introduction

Kidney transplantation with a graft from a living kidney donor (LKD) is the preferred treatment for most patients with end-stage kidney disease (ESKD) [1]. Due to superior outcomes for the transplant patient [2] and donor organ shortages, living kidney donation has become an important part of many transplant programs worldwide [1, 3]. The health outcomes of LKDs are favorable when compared with the general population [4], but when compared with selected non-donors, donors may have increased risk of hypertension, cardiovascular disease, ESKD and mortality [5–9]. This underscores the importance of evaluating the potential LKDs to ensure the safety of the donor and the quality of the transplanted graft. For the evaluation of LKDs, national and international guidelines exist [10–13], but little is known about their use in clinical practice. In 2020, a survey on LKD practices in the United States was published [14], which revealed ample variation in LKD selection practices between centers. While this survey was, in fact, the third one to be conducted in the United States since 1995, no similar initiative has been conducted in Europe. Here, we report the results of the first survey on LKD kidney function measurement, donor risk assessment, and follow-up practices in Europe.

## Materials and Methods

### Design of the Questionnaire

We used an online questionnaire to collect information on measurement of LKD kidney function, donor risk assessment, and post-donation follow-up practices in Europe. The questionnaire was administered to all relevant transplant professionals involved in the evaluation and/or follow-up of LKDs. Topics of the questionnaire were based on the 2017 evaluation of US donor practices [14] and were evaluated by the DESCaRTES working group of the European Renal Association (ERA) and EKITA working group of the European Society for Organ Transplantation (ESOT). Questions were entered, removed, or adapted in multiple rounds of discussion using the process of content validity through expert review. After agreement with the author group, the survey was tested by 10 transplant professionals (four nephrologists, four surgeons, and two clinical researchers in the field of kidney transplantation). The survey was designed, distributed and managed using REDCap electronic data capture tools hosted at the University Medical Center Groningen [15, 16].

The survey consists of 40–54 branched questions, with the number depending on previous answers. An overview of all the questions is provided in Supplementary Table S1. The questions were divided into five sections. The first section consists of questions on the center’s LKD program in general, the second section concerns kidney function evaluation, the third section is about LKD risk assessment, the fourth section is on follow-up practices, and the final section concerns data collection practices.

### Distribution of the Questionnaire

A link to the survey, accompanied by an introductory e-mail was dispatched to members of the DESCaRTES and EKITA working groups, who contacted members directly from their networks and asked them to forward the invitation for the questionnaire to relevant transplant professionals in their field. N = 125 complete responses were received, covering approximately 45% of European transplant centers (ESOT YPT Map of active European transplant centers, accessed at https://esot.org/map/). All respondents were invited to be recorded as collaborators in the final publication of the questionnaire.

Data are reported as percentages for all relevant questionnaire items. When a question has a numerical outcome, the median [25th; 75th percentile] is given. The transplant region of all respondent centers (Eurotransplant, Scandiatransplant, Southern Alliance or “Other,” including the United Kingdom and Turkey) was identified. Non-parametric tests were used to compare differences in responses between the centers (Kruskal-Wallis for continuous variables, Chi-squared test for categorical variables). A *p*-value of <0.05 was considered statistically significant. SPSS Statistics V23 (IBM, Armonk, United States), GraphPad Prism 8 (GraphPad Software, California, United States), and Microsoft Excel build 2406 (Microsoft, Redmont, United States) were used for data analyses and presentation.

## Results

### General Characteristics

**FIGURE 1:**
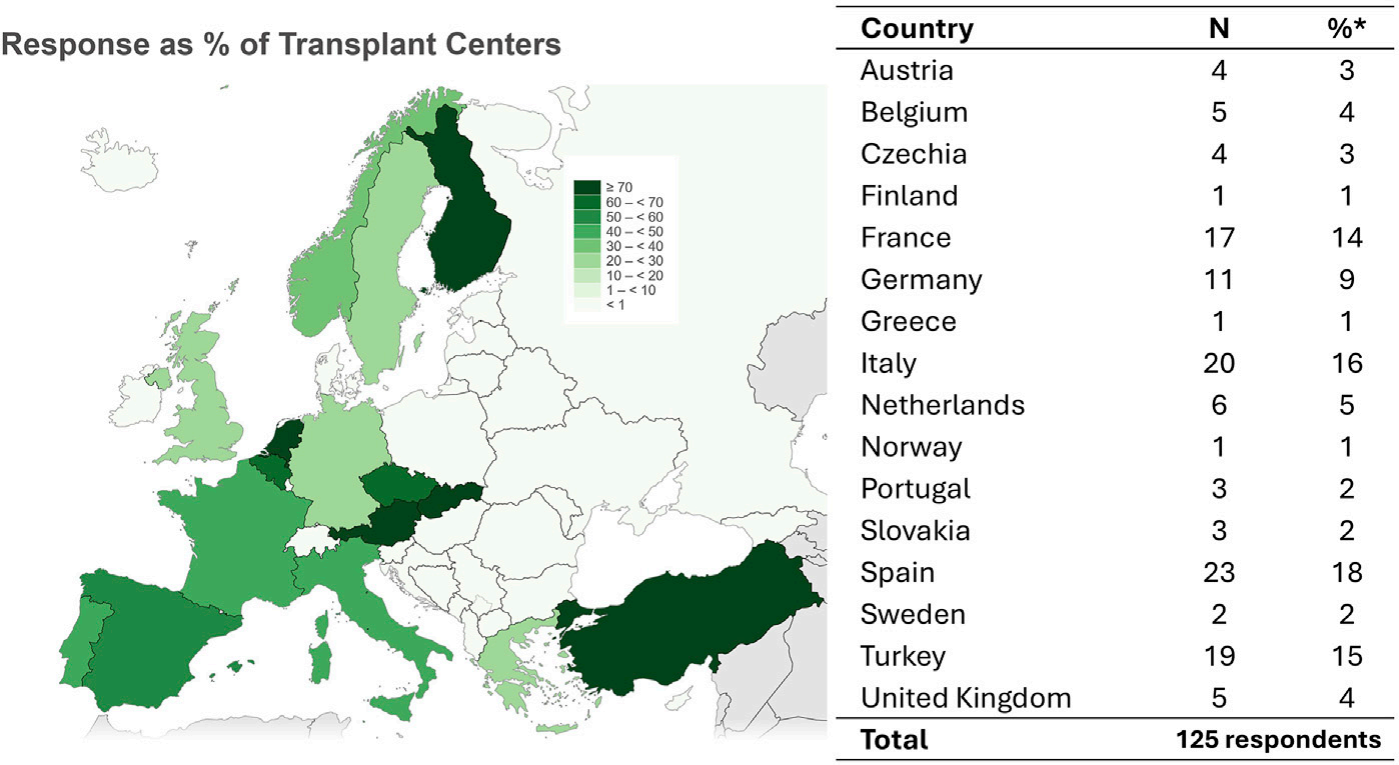
Overview of respondents. Overview of respondents as percentage of transplant centers per country (left) and absolute numbers with percentage of all reponses (right). Source: ESOT YPT Map of active European transplant centers (accessed at https://esot.org/map/). Created using IMAGE Interactive Map generator (accessed at: https://gisco-services.ec.europa.eu/image/screen/home).

We collected data from 125 respondents of 124 transplant centers, representing 45% of European transplant centers (Figure 1). Of all respondents, n = 112 (90%) were nephrologists, n = 10 (8%) were surgeons, and n = 3 (2%) were other transplant practitioners (e.g., specialized nurses). Respondents represented n = 16 countries, screening an estimated combined number of 8141 potential LKDs per year and performing about 3700 LKD transplantations per year in the last 5 years.

**FIGURE 2:**
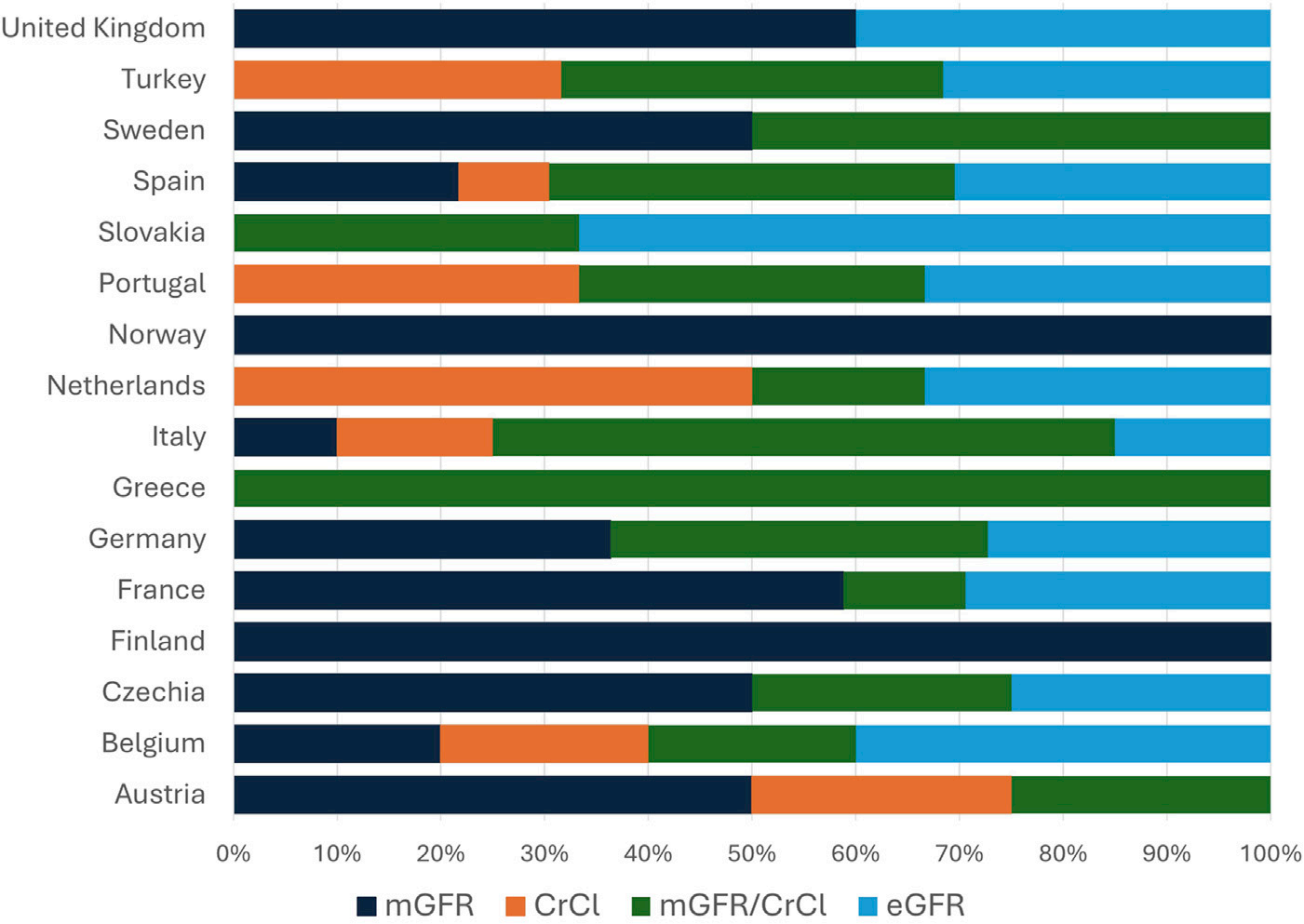
Transplant professionals involved in living kidney donor decision making. Overview of transplant professionals routinely involved in the selection of living kidney donation, expressed as percentage of all respondents (n = 125). When “Other” was selected, respondents were asked to specify: 2 (2%) respondents answered, “social worker,” 1 (1%) respondent answered “pharmacist,” 1 (1%) respondent answered “vascular surgeon” and 1 (1%) respondent answered “healthcare ethics committee” to be routinely involved in the selection of living kidney donors.

The screening of potential LKDs takes a median of 10 [2; 48] hours, and the entire process takes 30 [8; 60] days, either as an inpatient evaluation (21%), outpatient evaluation (54%) or both, according to donor choice (25%). An overview of all professionals involved is shown in Figure 2. Most centers base their practice on guidelines; n = 62 (50%) used the KDIGO guidelines, n = 9 (7%) use the BTS guideline, n = 30 (24%) use both and n = 24 (19%) use local guidelines or a combination of guidelines. Most potential LKDs are asked for written informed consent for nephrectomy at the screening (30%), after being approved (36%), before surgery (19%) or repeatedly (10%). Five percent of centers do not routinely ask for written informed consent for donation. Most centers register LKD data locally (24%) or in national databases/registries (26%). 41% of centers register data in both, while 9% do not register data. In 65% of centers donors provide written informed consent for the registration of their data.

### Evaluation of Kidney Function

**FIGURE 3:**
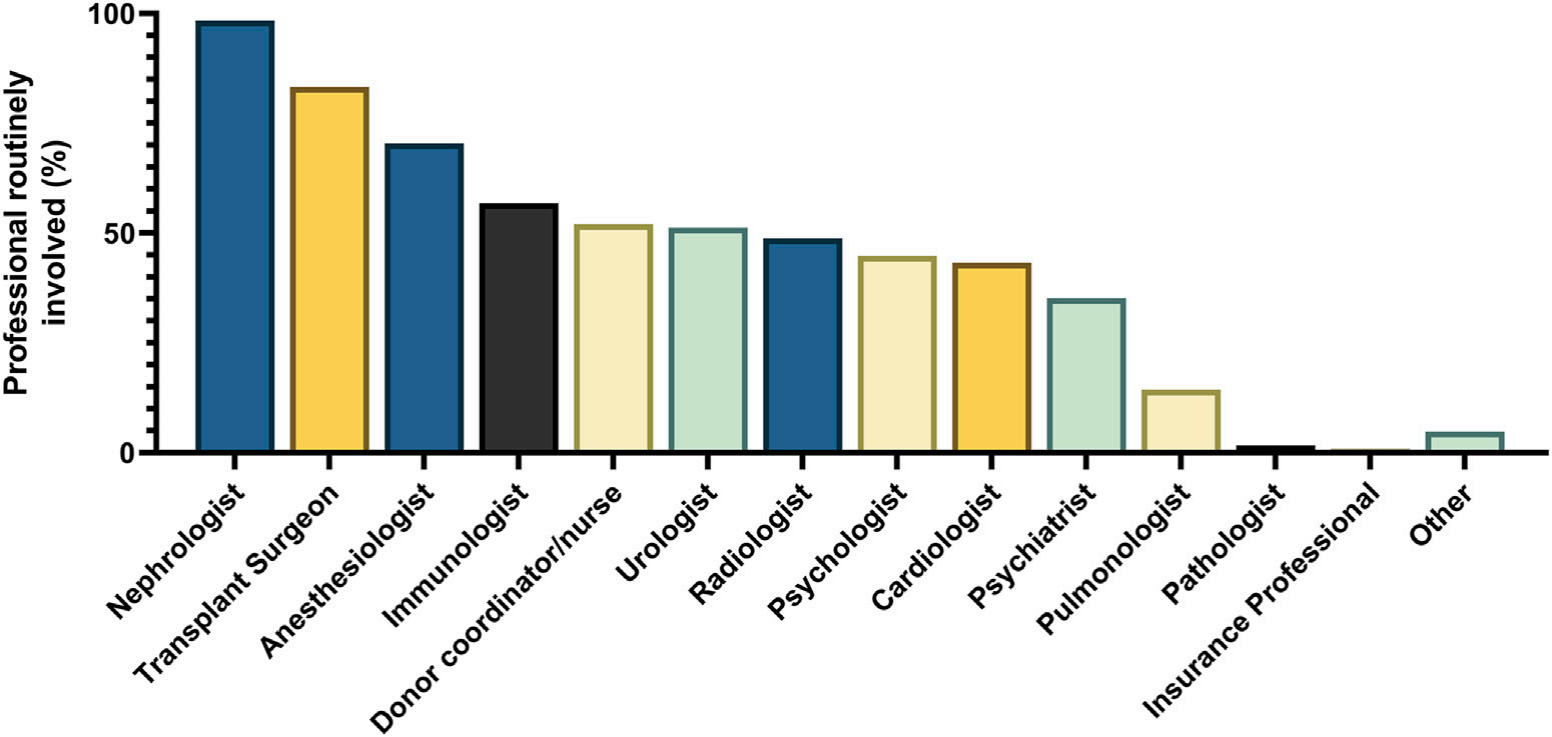
Kidney function assessment per country. Overview of routinely used tests for decision-making regarding kidney function of a potential LKD. Centers were asked which test they mainly use for decision-making: measured GFR (dark blue), 24-hour creatinine clearance (orange), a combination of these (green) or estimated GFR (light blue). Answers are expressed as percentage of all respondents (n = 125).

For the evaluation of kidney function, most centers use the Chronic Kidney Disease Epidemiology Collaboration (CKD-EPI)-equation (n = 70, 56%), while a minority use the Modification of Diet in Renal Disease (MDRD)-equation (n = 5, 4%), the European Kidney Function Consortium (EKFC)-equation (n = 1, 1%) or the 24-hour creatinine clearance (CrCl, n = 43, 34%) and n = 6 (5%) did not specify the test. N = 51 (41%) centers use a combination of creatinine and cystatin C for GFR estimation, while n = 4 (3%) centers use cystatin C without creatinine. Centers not using cystatin C indicate a lack of availability (n = 20), no perceived added value (n = 21) or costs (n = 7) as arguments for not using cystatin C. N = 88 (70%) use measured GFR (mGFR, using an exogenous marker) in their practice, of which n = 60 (68%) routinely perform mGFR. An overview of practices regarding mGFR is shown in Table 1. Most centers use an age-dependent GFR threshold to select LKDs (n = 80, 64%). Centers using a fixed threshold most often use 80 mL/min/1.73 m^2^ (n = 33, 26%). Centers in the United Kingdom, Norway, Spain, Germany, France, Finland, Czechia and Austria more frequently use mGFR-based screening. The use of CrCl is most common in Turkey, Portugal, the Netherlands and Italy (Figure 3). When differences between kidney sizes are found in imaging performed as part of the anatomical evaluation of the donor candidate, most centers perform split kidney function testing (n = 88, 70%).

**TABLE 1:** Practices regarding measured GFR in living kidney donor candidates.

Variable	Centers
Use of mGFR, n (%)	
Incidentally	28 (22%)
Routinely	60 (48%)
Never	37 (30%)
Tracer used, n (% of 88 centers)	
Plasma 99mTC-DTPA clearance	52 (59%)
Urinary 99mTC-DTPA clearance	5 (6%)
Plasma iohexol clearance	19 (22%)
Urinary iohexol clearance	3 (3%)
Plasma 125I-iothalamate clearance	2 (2%)
Urinary 125I-iothalamate clearance	3 (3%)
Other	4 (5%)
Indexation of mGFR, n (% of 88 centers)	
Indexed for BSA	52 (59%)
Unindexed	16 (20%)
Use both	17 (19%)
No answer	3 (2%)
Use of confirmatory testing in decision-making	
mGFR and CrCl	42 (34%)
Mainly mGFR	32 (26%)
Only CrCl	17 (14%)
mGFR dependent on eGFR	9 (7%)
CrCl dependent on eGFR	14 (11%)
eGFR only	11 (9%)

mGFR, measured Glomerular Filtration Rate; BSA, Body Surface Area; CrCl, 24-hour creatinine clearance; eGFR, estimated Glomerular Filtration Rate.

### Assessment of LKD Risks

Most centers have a lower age limit of 18 years old (n = 60, 53%), with the range of lower age-limits between 18 and 40 years old. 15 centers (23%) do not have a lower age limit. Most centers (n = 79, 63%) do not use an upper age limit. Centers with upper limits use 70 (9%), 75 (13%) or 80 (10%) years of age. BMI cut-offs of ≥30 (39%) or ≥35 kg/m^2^ (42%) are used to reject LKD candidates. Most centers offer weight loss interventions to overweight candidates (74%); responders provide dietary support (67%), exercise therapy/training support (27%), endocrinological evaluation and/or medication (23%), or bariatric surgery (11%).

To assess the risk for diabetes, centers either use an oral glucose tolerance-test (OGTT) in all donor candidates (25%), in candidates with elevated fasting glucose (65%), elevated HbA1c (52%), a family history of diabetes (33%) or obesity (41%). A minority of centers perform an OGTT in potential LKDs with hypertension (6%), dyslipidemia (2%) or isolated microalbumuria without other abnormalities (16%). Centers usually reject donor candidates with a HbA1c ≥ 53 mmol/mol or 7% (46%), fasting glucose above 7 mmol/L or 126 mg/dL (57%), or glucose after an OGTT ≥11.1 mmol/L or 199 mg/dL (59%). 10% of centers reject candidates with gestational diabetes, and 11% of centers reject younger candidates if they have ‘pre-diabetes’, while some respondents (n = 9) indicated that this decision depends on the entire risk profile.

Blood pressure is usually assessed using automated or non-automated office blood pressure measurements (46% and 26%, respectively), while 24-hour ambulant blood pressure measurements are performed in 34% of centers. Almost all centers reject donor candidates with uncontrolled hypertension and/or signs of end-organ damage during screening (91%), 19% reject candidates using ≥2 antihypertensives and 78% candidates with ≥3 antihypertensives. Persistent borderline hypertension, without end-organ damage, was not indicated as reason to reject candidates.

**FIGURE 4:**
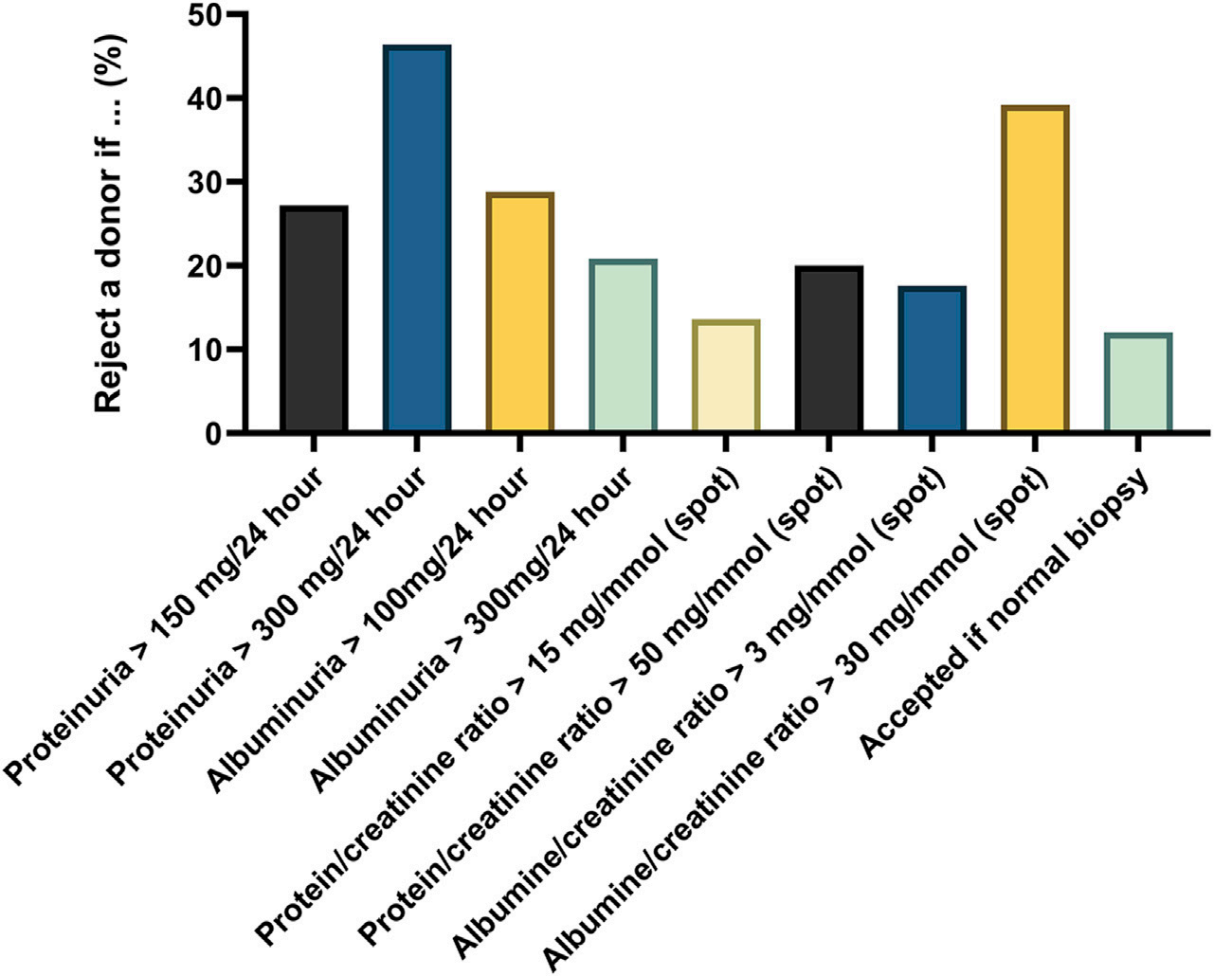
Overview of decision-making regarding proteinuria and albuminuria. Overview of decision-making regarding proteinuria/albuminuria in 24-hour urine and/or spot urines. Centers were asked which of the answers best represents their practice regarding the exclusion of donors with proteinuria. Multiple answers could be given. Answers are expressed as percentage of all respondents (n = 125). 12% of centers would accept donors with any proteinuria/albuminuria if they have a normal biopsy result.

38% of donor candidates undergo both spot urine and 24-hour urine test for proteinuria or albuminuria, while a minority undergoes 24-hour proteinuria/albuminuria testing (18% and 10%, respectively) or spot urine testing for proteinuria/albuminuria (9% and 24%, respectively). An overview of proteinuria-related decision-making is shown in Figure 4. 28 (22%) of centers base decision-making only on proteinuria (either 24-hour urine, spot urine or both).

Donor candidates with persistent isolated microscopic haematuria are mostly excluded in 5% of centers, while 42% of centers only exclude when urine sediment indicates a glomerular cause. Candidates with persistent isolated microscopic haematuria are usually accepted when they have no abnormalities in urological evaluation (15%), kidney biopsy (16%) or both (57%). 62% of centers do not perform kidney biopsies.

Donor candidates with a positive family history of Autosomal Dominant Polycystic Kidney Disease (ADPKD) are sometimes rejected outright (n = 4, 3%), depending on their age (n = 24, 19%), but most often receive additional testing with MRI (n = 28, 22%), ultrasound (n = 51, 41%) or MRI/ultrasound imaging depending on their age (n = 49, 39%) while some centers (n = 11, 9%) perform both imaging techniques. PKD-mutation analysis is performed in most of LKD candidates with a positive family history (n = 67, 54%).

27% of centers routinely perform ApoL1 testing for potential LKDs with African ancestry. For 11% of centers a high-risk ApoL1 genotype, when known, is a contra-indication for kidney donation.

2% of centers reject potential LKDs with any kidney stone, regardless of size or risk profile. Most centers accept donor candidates with a history of nephrolithiasis if no stones are present, the 24-hour urine profile is low-risk (36%) or when low-risk and stone-related symptoms were >5 years ago (29%). 29% of centers reject candidates with a history of bilateral stones.

NSAID use is accepted in 19% of centers when candidates are otherwise healthy. NSAID use is also accepted when a donor candidate has a rheumatological disease (2%), the use is infrequent (7%). 8% of centers accept some types of NSAIDs, while 61% of centers ask donors to stop NSAIDs completely. Smoking is a contra-indication for kidney donation in 3% of centers, whereas it is accepted (but strongly discouraged) in 78% of centers. Some centers ask LKD candidates to stop smoking 4 weeks before surgery, either with documentation of smoking-cessation (e.g., cotinine measurement, 3%) or without (16%).

A majority of centers do not routinely use online risk calculators to estimate lifetime risk of end-stage kidney disease (54%), 22% use the ESKD Risk Tool by Grams et al. [17] routinely and 22% for selected candidates. 2% use a different risk tool. Most centers do not use a fixed threshold for lifetime end-stage kidney disease in the donors, but rather an individualised risk leniency (57% use individualized thresholds, 1% report a threshold of 10%, 6% report a threshold of 5%, 5% report a threshold of 3% and 11% report a threshold of 1%). The remaining 21% do not use risk thresholds.

### Follow-Up of LKDs

Most centers (n = 97, 83%) routinely offer lifetime follow-up of donors. In centers with living donor follow-up, most LKDs receive a follow-up visit every year (90%) or every 2–4 years (10%). Follow-up generally consists of blood pressure checks (98%), 24-hour urinalysis (34%), spot urine analysis (75%), eGFR (94%), CrCl (15%), mGFR (18%), blood tests (83%), body composition measurements (67%) and/or a medication review (70%). Psychosocial counselling is offered in 20%. Follow-up is mostly organized by nephrologists (82%), general practitioners (8%) or transplant surgeons (7%). Follow-up involves out-of-pocket payment for travel expenses in 6% of centers and all post operative care in 1% of centers. 3% of centers indicate that follow-up is not always performed.

### Consensual and Controversial Practices

**FIGURE 5:**
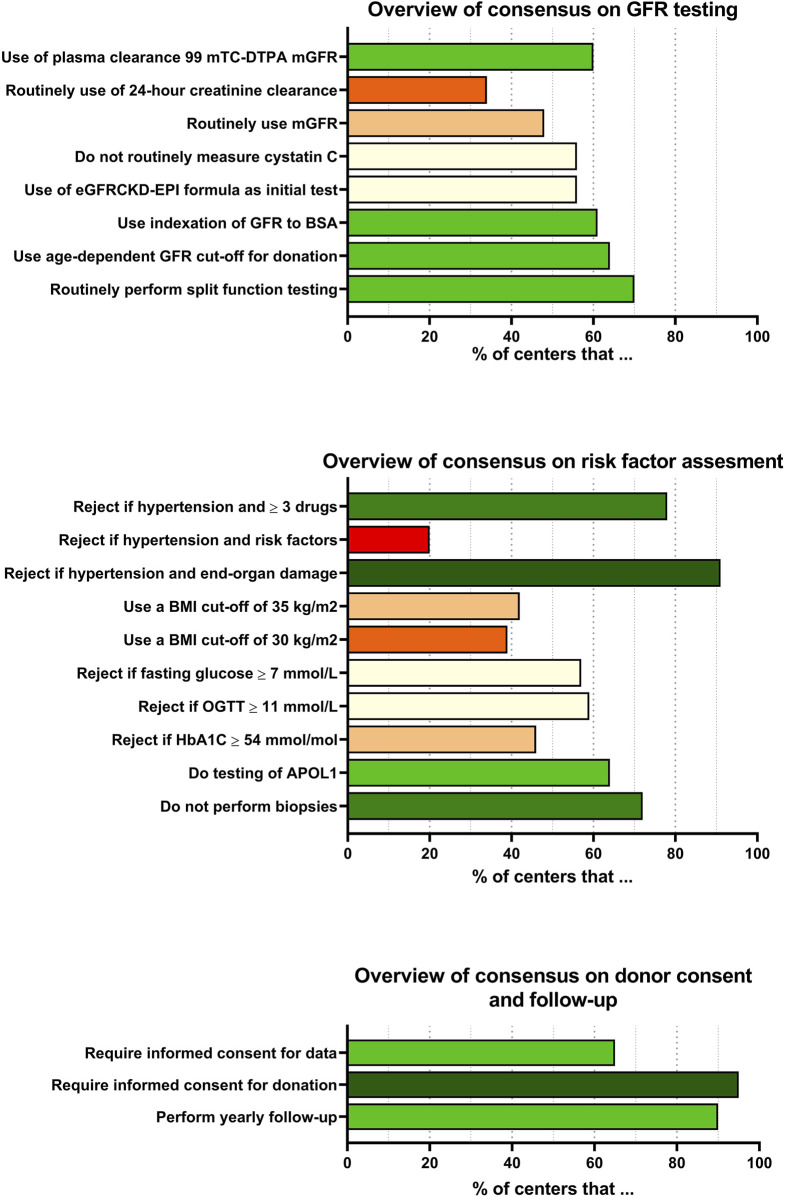
Consensus overview of questionnaire items. Overview of rate of consensus on various questionnaire items. When multiple answers were possible, the most common answer is shown in the graph.

The highest consensus among centers was in the requirement of informed consent for kidney donation, decision making around hypertension, the exclusion of donor candidates <18 years of age and the use of routine (mostly annual) follow-up after kidney donation. Practices with low consensus include the use of kidney function testing, the routine use of CrCl and the acceptance policy of donor candidates with nephrolithiasis. Also, centers differ in assessment of albuminuria, use of cystatin C and BMI cut-off values. An overview of the consensus of all questionnaire items is provided in Figure 5.

### Differences Between Transplant Regions

N = 29 (23%) of the respondents are part of Eurotransplant (ET), n = 4 (3%) of Scandiatransplant (ST), 67 (54%) of the Southern Alliance (SA), and 25 (20%) of other transplant regions. A detailed overview of questionnaire responses per transplant region can be found in Supplementary Table S2 for general characteristics, Supplementary Table S3 for kidney function assessment, Supplementary Table S4 for risk assessment and Supplementary Table S5 for donor follow-up. The number of LKD transplantations differed between respondents from the four identified transplant regions, with Scandiatransplant (median 36/year/center) performing the most LKD transplantations per center (P < 0.001, Supplementary Table S2). No differences were found when comparing the use of mGFR (P = 0.06), the use of mGFR tracer (p = 0.40) or GFR indexing (p = 0.34) or confirmatory testing (P = 0.57; Supplementary Table S3). Scandiatransplant more often performs OGTTs (P = 0.045, Supplementary Table S4), but no other differences in glucose testing were found. No differences were found between the regions regarding BMI cut-offs, but there were differences in weight loss interventions offered to LKD candidates (offered in 52% for ET, 25% for ST, 85% for SA, P < 0.001), with also more dietary interventions offered in the SA-region (45% vs. 0% vs. 78%, P < 0.001). No significant differences were found regarding ADPKD testing, nephrolithiasis, and haematuria testing (P > 0.05 for all analyses). Centers in the ET-region more often reject donors with a protein/creatinine ratio of >50 mg/mmol when no other abnormalities are present (P = 0.03), and centers in the ST- and SA-regions more often reject donors with an albumin/creatinine ratio >3 mg/mmol (P < 0.001). The ET-region more often accepts candidates with proteinuria if they have no abnormalities on biopsy (P = 0.03). Only in the ET-region do centers exclude smokers from donation (14% vs. 0% in other regions). The intensity, specialty in charge of follow-up and medical items part of follow-up differ per transplant region (Supplementary Table S5).

## Discussion

In this study, we show differences in the evaluation, selection and follow-up practices for LKDs across Europe. We report marked differences in the use of confirmatory kidney function testing (eGFR, creatinine/cystatin C, mGFR, creatinine clearance), albuminuria assessment, and the acceptance policy of donors with nephrolithiasis. These results point to opportunities for harmonization and future studies.

High standards for the acceptance of LKD candidates is paramount for ensuring the safety of LKDs and improving the quality of the transplanted graft. Although national and international guidelines exist for LKD evaluation (Table 2), our study highlights the differences in guideline application across Europe. Consistent with guideline recommendations, all centers have a dedicated team for the evaluation of LKD candidates, although the professionals involved in this team differ. In line with recommendations, centers obtain informed consent for donation, although consent for data use is inconsistent.

**TABLE 2:** Overview of National and International guidelines for the selection of Living Kidney Donors [10].

Title	Year	Organization	Reach/Origin	Source
KDIGO Clinical Practice Guideline on the Evaluation and Care of Living Kidney Donors	2017	KDIGO	Global	[11]
BTS/RA Living Donor Kidney Transplantation Guidelines 2018	2018	BTS/RA	United Kingdom	[12]
Recommandations d’aide à la pratique clinique pour le don de rein du vivant	2023	Agence de la Biomédecine	France	[13]
European Renal Best Practice Guideline on kidney donor and recipient evaluation and perioperative care	2015	ERBP	Europe	[18]
Samenvatting van aanbevelingen in de Britse richtlijn “Living Donor Kidney Transplantation”	2020	Nederlandse Transplantatie Vereniging	Netherlands	[19]

The guidelines stated in Table 2 show differences in kidney function testing recommendations, which are also clear from the responses to our survey. Overall, an individualized assessment of kidney function is performed, but centers and particularly countries differ in their technique, for example, eGFR (creatinine, cystatin C or both), 24-hour urinary creatinine clearance or measured GFR for decision-making. In 2022, the DESCARTES working group of the ERA released a position paper, where they advocated an individualized (and age-dependent) GFR threshold and recommend mGFR for LKD assessment [20]. Most centers (68%) routinely perform mGFR in donor candidates, using multiple possible tracers. In light of challenges in the production of radioactive tracers, and a publication calling for standardization of mGFR from the European Kidney Function consortium [21], iohexol plasma clearance (currently used in 22% of respondents) may become more common. Guidelines advocate normalizing kidney function for body surface area [11], and we report similar normalization rates compared to the United States (71% BSA-normalization in our survey vs. 75% in the US) [14].

Most centers (63%) do not set an upper age limit for kidney donation, reflecting a change in guidelines over time. While perioperative risks are higher for older donors, lifetime risks for end-stage kidney disease will always be higher for younger donors, due to remaining life-span being longer [7, 8, 17]. Accordingly, several centers reported stricter selection in younger donors. While obesity is a well-known risk factor for adverse outcomes in donation [22, 23], the presence of obesity is handled differently across transplant-centers and regions: cut-offs for BMI of 30 kg/m^2^ or 35 kg/m^2^ are both used. The importance of a healthy weight for LKDs is recognized; 74% of centers offer weight-loss interventions for LKDs, most often in the Southern Alliance. However, weight-loss interventions vary greatly between respondents: 67% offer dietary support, 27% offer exercise therapy/training, 23% offer endocrinological evaluation/medication and 11% offer bariatric surgery. While research on bariatric surgery in future LKDs is expanding [24, 25], secondary hyperoxaluria from bariatric surgery and corresponding nephrolithiasis/nephrocalcinosis are risk factors for CKD [26, 27]. If bariatric surgery is necessary for LKD candidates, sleeve gastrectomy reduces hyperoxaluria risk compared to Roux-en-Y bypass [28].

While risks of diabetes and hypertension are differently assessed between respondents, there is a consensus on the acceptance of LKD candidates with these comorbidities. In line with guidelines, candidates with uncontrolled hypertension and/or with signs of end-organ damage, are not accepted for donation. Candidates with diabetes are also excluded from donation. In line with the KDIGO guidelines, most centers reject donors with an abnormal OGTT, HbA1c, or fasting glucose. Guidelines differ in their recommendations on proteinuria testing: the KDIGO guideline advise using albuminuria and not proteinuria, because of standardization issues and evidence about albuminuria as an independent risk factor. The French guideline underscores this (grade B level of evidence), while the British Transplant Society guideline considers the measurement of total protein in the urine to be an acceptable alternative (grade A1 level of evidence). This is reflected in the answers to our survey, where decision-making is based on both, with most centers performing 24-hour assessment of protein/albumin excretion. Hematuria can be acceptable for LKD candidates, if no other abnormalities are found on urinalysis, urological evaluation and/or kidney biopsy. Candidates with persistent asymptomatic hematuria are rejected in a minority of centers, in line with most guidelines. Not all centers perform kidney biopsies in donors with hematuria, as recommended in the BTS guideline and suggested in the French/KDIGO guidelines [12, 13, 29]. Acceptance of LKD candidates with nephrolithiasis varies although most centers accept candidates with historical stone disease provided the recurrence risk is deemed low, in line with the guidelines and supported by the literature [30]. ApoL1 testing for donors with African ancestry is routinely performed in a minority (27%), while it is considered in the risk profile when known. In comparison, in the 2017 survey in the United States, 13% of respondents routinely performs ApoL1 genotyping and 32% perform this for selected candidates [14]. Interestingly, NSAID use is acceptable in 19% of centers and conditionally accepted in another 17%, in line with data from the US [14]. While smoking is an important modifiable risk factor for cardiovascular and kidney disease [17], it is not generally a contra-indication for kidney donation. Some centers ask donors to stop 4 weeks before the surgery, possibly because of the increased risk of complications found in non-donation surgery [31].

A minority use the end-stage kidney disease (ESKD) Risk Tool by Grams et al. either routinely or for selected candidates (45%) [17], which is slightly less than in the US survey [14]. Most centers using thresholds for ESKD risk leniency reported individualized thresholds, or no numerical threshold at all. Limitations of the ESKD Risk tool and other calculators include lack of validation outside the cohorts they were developed in (a non-donor US population), and a lack of consensus on relevant thresholds for individual candidates [20]. Also, long-term risk for ESKD is impossible to capture from baseline data in younger donors [32, 33]. Use of an ESKD Risk tool may therefore falsely re-assure donors and clinicians of limited risks. In younger donors, lifelong follow-up is of special importance, even if they have an apparent low risk of ESKD [20].

Long-term follow-up of kidney donors is considered necessary and often mandatory, although specifics vary between centers. While 17% of centers do not promote lifetime follow-up, 10% organize follow-up every 2–4 years. Follow-up is mainly managed by the nephrologist but may be organized by general practitioners, most often in the Eurotransplant region, likely due to the local practice and reimbursement policies. Follow-up generally includes a medication review, cardiovascular risk assessment, spot urine analysis and blood tests. A minority of centers also incorporate psychosocial counselling.

This study has several limitations. While it offers broad representation across Europe (Figure 2), the Eastern part of Europe is underrepresented. This limitation may be cause by not having sufficient contact details in this area and could indicate more necessity for outreach by European transplant professionals and organisations. Survey fatigue could also have been a reason for a limited response rate in some areas. We aimed to limit this, by choosing one respondent for a transplant center to answer, which could induce bias itself: The questionnaire was designed to identify practice variation between centers, rather than between individuals within centers. The questionnaire format is subject to social desirability bias and recall bias. Also, statistical analyses comparing transplant regions were limited by power and multiple testing (increasing the chance of type I error). Donor evaluation and follow-up decisions are often individualized and may not apply uniformly across cases, a complexity not fully captured by questionnaires. When developing the questions, we specifically attempted to recognize this caveat. Our survey was inspired by the initiative from the United States to evaluate the LKD practices [14], but results between the US and Europe cannot be compared directly because ours was more recent (2023 vs. 2017) and the healthcare systems in Europe and the US differ [34]. Our survey benefits from a high response rate (Figure 1), a wide range of assessed topics and the addition of data on follow-up practices.

The current study provides a snapshot of current living kidney donor practices across Europe and can help to inform healthcare professionals on prevalent practices. Our findings may support the development of healthcare policies aimed at improving the quality of LKD information, selection and follow-up. Future studies should focus on the role of cultural, social or logistical factors in living kidney donor practices, for example, on how the availability of resources influencing kidney function testing. Our results underscore the importance of harmonization of living donor care using evidence-based practice. We also advocate for the establishment of a European registry of LKD outcomes to further study LKD practices and outcomes [35, 36].

In conclusion, this is the first study on practices in the evaluation, selection and follow-up of LDKs in Europe. The selection of LKDs is a balancing act between the benefits for the donor- and the recipient on one hand, and short-term and long-term risks of donor nephrectomy on the other hand [33]. Our study identified several areas with considerable heterogeneity between centers and regions, especially in confirmatory kidney function testing, use of 24- hour creatinine clearance and the acceptance policy of donors with nephrolithiasis. Heterogeneity was also apparent in the assessment of albuminuria, use of cystatin C and BMI cut-offs. This heterogeneity can be used as a basis for future studies and should serve to dynamically inform professionals, help design healthcare policies and improve the overall quality of information, selection and follow-up of living donors. We, therefore, support harmonization of living donor management using evidence-based practice and call for a European registry of LKD outcomes [35, 36].

## Data Availability

The raw data supporting the conclusions of this article will be made available by the authors, without undue reservation.

## Group Member of Study Collaborators

Giuseppe Tisone, Azienda Ospedaliera G. Brotzu, Cagliari, Italy; Hamad Dheir, Division of Nephrology, Faculty of Medicine, Sakarya University, Turkey; Francesc J. Moreso, Nephrology Departments, Hospital Universitari Vall d’Hebron, Barcelona, Spain; Massimiliano Veroux, Organ Transplant Unit, Department of Surgical and Medical Sciences and Advanced Technologies, University Hospital of Catania, Catania, Italy; Sophie Caillard, Department of Nephrology, Dialysis and Transplantation, Strasbourg University, Strasbourg, France; Cihat B. Sayın, Department of Internal Medicine, Division of Nephrology, Başkent University Faculty of Medicine, Ankara, Turkey; Sheila Cabello, Nephrology Department, Hospital Universitario Son Espases, Mallorca, Spain; Luigino Boschiero, Kidney Transplant Surgery Division, Verona University Hospital, Verona, Italy; Ercan Turkmen, Department of Nephrology, Ondokuz Mayis University Faculty of Medicine, Samsun, Turkey; Juan Carlos Ruiz, Nephrology Department, Marqués de Valdecilla University Hospital-IDIVAL, University of Cantabria, Spain; Simona Simone, Renal, Dialysis and Transplantation Unit, Department of Emergency and Organ Transplantation, University of Bari, Bari, Italy; Arnoud Del Bello, Department of Nephrology and Organ Transplantation, CHU de Toulouse, Toulouse, France; Giuseppe Grandaliano, Nephrology Unit, Department of Medical and Surgical Sciences, Fondazione Policlinico Universitario A. Gemelli IRCCS, Rome, Italy; Andrea Ranghino, Nephrology, Dialysis and Renal Transplantation Unit, Azienda Ospedaliera Universitaria Ospedali Riuniti Umberto I, Lancisi, Salesi of Ancona, Ancona, Italy; Giorgia Comai, Department of Experimental, Diagnostic and Specialty Medicine (DIMES)-Nephrology, Dialysis and Renal Transplant Unit, St. Orsola-Malpighi Hospital, University of Bologna, Bologna, Italy; Francesco Pisani, General and Transplant Surgery Department, University of L'Aquila, 67100 L'Aquila, Italy; Fabio Vistoly, Department of Oncology and Transplant Surgery, University of Pisa, Pisa, Italy; Bruno Watschinger, Department of Internal Medicine III, Nephrology, Medical University Vienna/AKH Wien, Vienna, Austria; Aida Larti, Nephrology Unit, Careggi University Hospital, Florence, Italy; Rachel Hellemans, Department of Nephrology/Hypertension, Antwerp, University Hospital, Edegem, Belgium; Ülkem Çakir Department of Nephrology, Acibadem University School of Medicine, Istanbul, Turkey; Gürsel Yildiz, Department of Nephrology, Faculty of Medicine, Cumhuriyet University, Sivas, Turkey; Aygul Celtik, Division of Nephrology, Department of Internal Medicine, School of Medicine, Ege University, Bornova, Izmir, Turkey; Jan-Stephan F. Sanders, Department of Internal Medicine, Division of Nephrology, University Medical Center Groningen and University of Groningen, Groningen, the Netherlands; Auxiliadora Mazuecos, Department of Nephrology, Hospital del Puerta del Mar, Cadiz, Spain; Gabriel Bernal, Nephrology Department, Hospital Universitario Virgen del Rocío, Seville, Spain; Pavlína Richtrová, Department of Internal Medicine I, Faculty of Medicine in Pilsen, Charles University, and Teaching Hospital in Pilsen, Plzen, Czech Republic; Zuzana Žilinská, Urological Clinic and Center for Kidney Transplantation, University Hospital Bratislava and Faculty of Medicine, Comenius University in Bratislava, Bratislava, Slovakia; Paloma Leticia Martin-Moreno, Department of Nephrology, Clinica Universidad de Navarra, Navarra Institute for Health Research (IdiSNA), Pamplona, Spain; La Salete Martins, Department of Nephrology, Centro Hospitalar Universitário de Santo António (CHUdSA), Porto, Portugal; Bengt von Zur-Mühlen, Department of Surgical Sciences, Section of Transplantation Surgery, Uppsala University, Uppsala, Sweden; Smaragdi Marinaki, Clinic of Nephrology and Renal Transplantation, Laiko Hospital, Athens Greece; Stefan Reuter, Department of Medicine D, Division of General Internal Medicine, Nephrology and Rheumatology, University Hospital Münster, Münster, Germany; Ulrich Pein, Martin Luther University Halle-Wittenberg, Department of Internal Medicine II, Halle, Germany; Dirk L. Stippel, Department of General, Visceral, Cancer and Transplant Surgery, University Hospital Cologne, Cologne, Germany; Gabriel Choukroun, Nephrology Dialysis and Transplantation Department, Amiens University Hospital, Amiens, France; Jean-Philippe Rerolle, Service de transplantation rénale, CHU Dupuytren, Limoges, France; Claire Tinel, Department of Nephrology and Kidney Transplantation, Dijon University Hospital, Dijon, France; Ana González-Rinne, Nephrology Department, Hospital Universitario de Canarias, Tenerife, Spain; Emilio Rodrigo, Nephrology Department, Hospital Universitario Marqués de Valdecilla/IDIVAL, Santander, Spain; Karel Krejčí, University Hospital Olomouc and Palacký University Olomouc, Faculty of Medicine and Dentistry, Department of Internal Medicine III – Nephrology, Rheumatology and Endocrinology, Olomouc, Czech Republic; François Provot, Department of Nephrology, Lille University Hospital, Lille, France; Vincent Vuiblet, Service de Pathologie, Institut d'Intelligence Artificielle en Santé, CHU de Reims et Université de Reims Champagne Ardenne, Reims, France; Maarten Naesens, Nephrology and Renal Transplantation Research Group, Department of Microbiology, Immunology and Transplantation, KU Leuven, Leuven, Belgium; Department of Nephrology and Renal Transplantation, University Hospitals Leuven, Leuven, Belgium; Umberto Maggiore, Department of Medicine and Surgery, Nephrology Unit, Azienda Ospedaliera-Universitaria Parma, University of Parma, Parma, Italy; Lionel Couzi, Service de Néphrologie, Transplantation, Dialyse et Aphérèses, CHU de Bordeaux, Bordeaux, France; Michael Rudnicki, Department of Internal Medicine IV, Medical University of Innsbruck, Innsbruck, Austria; Anna Manonelles, Hospital Universitari de Bellvitge, Nephrology Department. L'Hospitalet de Llobregat, Spain; Biomedical Research Institute (IDIBELL), Hospital Duran i Reynals, Barcelona, Spain; Vladimir Hanzal, Department of Nephrology, Institute for Clinical and Experimental Medicine (IKEM), Prague, Czechia; Alex Gutiérrez-Dalmau Department of Nephrology, Hospital Universitario Miguel Servet, Zaragoza, Spain; Magali Giral, Institut de Transplantation Urologie Néphrologie (ITUN), CHU Nantes, Nantes, France; Carlos Jimenez, Hospital Universitario La Paz / Instituto de Investigación Hospital Universitario La Paz, Madrid, Spain; Ilkka Helanterä, Helsinki University Hospital and University of Helsinki, Transplantation and Liver Surgery Unit, Finland; Jessica Smolander, Department of Nephrology, Karolinska University Hospital and Division of Renal Medicine, Department of Clinical Science, Intervention and Technology (CLINTEC), Karolinska Institutet, Stockholm, Sweden; Anja S. Mühlfeld, Uniklinik RWTH Aachen, Aachen, Germany; Kai Lopau, University Hospital, Julius-Maximilians-University of Wuerzburg, Würzburg, Germany; Katalin Dittrich, Division of Pediatric Nephrology, University of Leipzig Medical Center, Leipzig, Germany; Gunilla Einecke, Department of Nephrology and Rheumatology, University Medical Center Göttingen, Göttingen, Germany; Claire Pitchford, Live Donor Team, Northen Care Alliance, Salford Royal NHS Foundation Trust, Salford, UK; Grahame Wood, Department of Nephrology, Salford Royal NHS Foundation Trust, Salford, UK; Natalia Ridao-Cano, Department of Nephrology, Hospital Clinico Universitario San Carlos, Madrid, Spain; Andrea Ambrosini, Renal Transplant Unit, Azienda Ospedaliera Ospedale di Circolo e Fondazione Macchi, Varese, Italy; Basar Aykent, Division of Nephrology, Department of Internal Medicine, Marmara University School of Medicine, Istanbul, Turkey; Cristina Jorge, Nephrology Department, Hospital de Santa Cruz – Centro Hospitalar de Lisboa Ocidental, Carnaxide, Portugal; Guido Garosi, Department of Medical Science, Nephrology, Dialysis and Transplantation Unit, University Hospital of Siena, 53100 Siena, Italy; Sabine Zitta, Division of Nephrology, Department of Internal Medicine, Medical University of Graz, Graz, Austria; Nicola Bossini, Unit of Nephrology, ASST Spedali Civili di Brescia, Brescia, Italy; Arjan D. van Zuilen, Department of Nephrology, University Medical Center, Utrecht, Utrecht, The Netherlands; Concetta Catalano, Department of Nephrology, Cliniques Universitaires de Bruxelles - Hôpital (CUB) Erasme, Brussels, BEL; Dilek Barutcu Atas, Division of Nephrology, Department of Internal Medicine, School of Medicine, Marmara University, Istanbul, Turkey; Ebru Sevinc Ok, Division of Nephrology, Ege University School of Medicine, Bornova, Izmir, Turkey; Ayse Serra Artan, Division of Nephrology, Department of Internal Medicine, Istanbul Faculty of Medicine, Istanbul University, Istanbul, Turkey; Meltem Gursu, Haseki Training and Research Hospital, Department of Nephrology, Istanbul, Turkey; Arwa Jalal Eddine, Department of Nephrology, Foch Hospital, Suresnes, France; Annelies E. de Weerd, Rotterdam Transplantation Institute, Department of Nephrology & Transplantation, Erasmus Medical Center, Rotterdam, The Netherlands; Julia Kanter, Department of Nephrology, Hospital Universitari Dr. Peset, Fundación para el Fomento de la Investigación Sanitaria y Biomédica de la Comunitat Valenciana, Valencia, Spain; Sultan Ozkurt, Eskişehir Osmangazi University, Faculty of Medicine, Department of Nephrology, Eskisehir, Turkey; Steven Van Laecke, Renal Division, Department of Internal Medicine, Ghent University Hospital, Ghent, Belgium; Nada Kanaan, Nephrology Division, Department of Internal Medicine, Cliniques Universitaires Saint-Luc, Brussels, Belgium; Marta Crespo, Department of Nephrology, Hospital del Mar, Barcelona, Spain; Carla Burballa, Department of Nephrology, Hospital del Mar, Barcelona, Spain; Huseyin Kocak, Department of Organ Transplantation, Akdeniz University Faculty of Medicine, Antalya, Turkey; Serpil Müge Değer, Dokuz Eylul University Faculty of Medicine, Department of Internal Medicine, Division of Nephrology, Izmir, Turkey; Frederike J. Bemelman, Department of Nephrology, Amsterdam University Medical Center, University of Amsterdam, Amsterdam, The Netherlands; Vera Paloschi, Clinic Unit of Regenerative Medicine and Organ Transplants, IRCCS Ospedale San Raffaele, Milan, Italy; Gaetano La Manna, Nephrology, Dialysis and Renal Transplant Unit, IRCCS-Azienda Ospedaliero-Universitaria di Bologna, Alma Mater Studiorum University of Bologna, Bologna, Italy; Mehmet Emin Demir, Department of Nephrology and Organ Transplantation, Faculty of Medicine, Yeni Yuzyil University, Istanbul, Turkey; Eva Lacková, Transplant Centre, University Hospital F.D. Roosevelta, Banska Bystrica, Slovak Republic; María Luisa Rodríguez Ferrero, Hospital General Universitario Gregorio Marañón, Madrid, Spain; Juliane Putz, Department of Urology, University Hospital Carl Gustav Carus, TU Dresden, Dresden, Germany; Vincent Pernin, Centre Hospitalier Universitaire de Lapeyronie, Département de Néphrologie Dialyse et Transplantation Rénale, Montpellier, France; Abdulmecit Yildiz, Department of Medicine, Division of Nephrology, Uludag University School of Medicine, Bursa, Turkey; Johan Noble, Centre Hospitalier Universitaire de Grenoble, La Tronche, France; Marie-Benedicte Matignon, Department of Nephrology and Transplantation, Hôpital Henri Mondor APHP, Creteil, France; Carme Facundo Molas, Nephrology Department, Fundación Puigvert, Barcelona, Spain; Ivana Dedinská, Department of Surgery and Transplantation Center, University Hospital Martin, Jessenius Medical Faculty of Comenius University, Martin, Slovakia; Michaela Matysková Kubišová, Charles University, Hradec Králové, Czech Republic.
