# Supplementary material for: Living Kidney Donation Practices in Europe: A Survey of DESCaRTES and EKITA Transplantation Working Groups
Source: Transpl Int. 2025 Jul 15;38:14802. doi: 10.3389/ti.2025.14802 (PMC12303855; doi:10.3389/ti.2025.14802)
Supplement: Supplementary file 1 [file Supplementaryfile1.docx]

***Supplementary data***

**
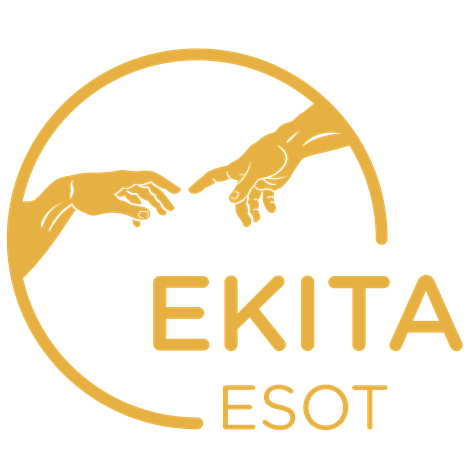
**
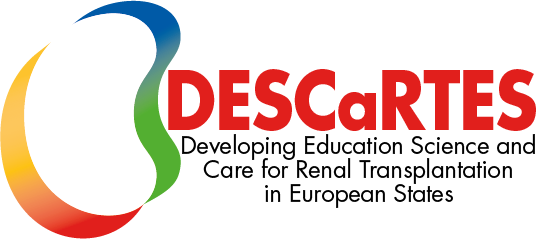


**Contents:**

**Table S2: General characteristics of living kidney donation per transplant region**

**Table S3: Kidney function assessment per transplant region**

**Table S4: Donor risk assessment per transplant region**

**Table S5: Donor follow-up practices per transplant region**

**Table S2: General characteristics of living kidney donation per transplant region**

|  | Region | | | |  |
| --- | --- | --- | --- | --- | --- |
| Questionnaire item | **Eurotransplant** | **Scandiatransplant** | **Southern Alliance** | **Other** | **P-value** |
| Number of centers | 29 (23%) | 4 (3%) | 67 (54%) | 25 (20%) |  |
| Number of transplantations per center, median [25^th^;75^th^] | 79 [ 50;136] | 185 [112;263] | 75 [54;110] | 50 [25;100] | 0.02 |
| Number of potential LKD evaluated per center, [25^th^;75^th^] | 35 [20;80] | 39 [16;65] | 30 [15;70] | 80 [45;100] | 0.005 |
| Number of LKD transplantations per center, median [25^th^;75^th^] | 15 [10;39] | 36 [30;53] | 10 [4;22] | 30 [21;60] | <0.001 |
| Duration of donor screening, hours, median [25^th^;75^th]^ | 15 [3;48] | 4 [2;27] | 7 [2;48] | 24 [6;72] | 0.30 |
| Duration of complete screening process, days median [25^th^;75^th^] | 30 [15;48] | 75 [44;90] | 30 [14;90] | 14 [5;30] | 0.02 |
| Professionals involved in donor screening*, n* (%)**  Anaesthesiologist  Cardiologist  Insurance professional  Nurse/coordinator  Immunologist  Nephrologist  Surgeon  Pathologist  Pulmonologist  Psychiatrist  Psychologist  Radiologist  Urologist  Other (health ethics, pharmacist, social worker) | 11 (40%)  8 (28%)  0 (0%)  10 (35%)  18 (62%)  28 (97%)  27 (93%)  0 (0%)  2 (7%)  3 (10%)  15 (52%)  9 (31%)  8 (28%)  3 (10%) | 1 (25%)  0 (0%)  0 (0%)  3 (75%)  1 (25%)  4 (100%)  4 (100%)  0 (0%)  0 (0%)  0 (0%)  1 (25%)  1 (25%)  0 (0%)  0 (0%) | 56 (84%)  29 (43%)  1 (1%)  32 (48%)  37 (55%)  67 (100%)  50 (75%)  1 (1%)  3 (4%)  24 (36%)  37 (55%)  35 (52%)  42 (63%)  2 (3%) | 20 (80%)  17 (68%)  0 (0%)  20 (80%)  15 (60%)  24 (96%)  12 (92%)  1 (4%)  13 (52%)  17 (68%)  3 (12%)  16 (64%)  14 (56%)  1 (4%) | <0.001  0.007  0.83  0.005  0.54  0.44  0.05  0.69  <0.001  <0.001  0.002  0.07  0.002  0.45 |
| Use of guidelines*, n* (%)  KDIGO guideline  BTS guideline  Both guidelines  Other/unspecified | 17 (59%)  1 (3%)  7 (24%)  4 (14%) | 1 (25%)  0 (0%)  0 (0%)  3 (75%) | 29 (43%)  4 (6%)  17 (25%)  17 (25%) | 15 (60%)  4 (16%)  6 (24%)  0 (0%) | 0.02 |
| Informed consent for donation*, n* (%)  Yes, at the start of the screening process  Yes, after being approved  Yes, before surgery  Yes, repeatedly  No | 12 (41%)  9 (31%)  4 (14%)  4 (14%)  0 (0%) | 1 (25%)  1 (25%)  2 (50%)  0 (0%)  0 (0%) | 18 (27%)  25 (37%)  10 (15%)  8 (12%)  6 (9%) | 7 (28%)  10 (40%)  8 (32%)  0 (0%)  0 (0%) | 0.19 |
| Registry of donor data*, n* (%)  Yes, locally  Yes*, n*ationally  Yes, both locally and nationally  No | 4 (14%)  8 (28%)  12 (41%)  5 (17%) | 0 (0%)  3 (75%)  1 (25%)  0 (0%) | 17 (25%)  17 (25%)  32 (48%)  1 (1%) | 9 (36%)  5 (20%)  6 (24%)  5 (20%) | 0.01 |
| Informed consent for data registry*, n* (%)  Yes  No | 20 (83%)  4 (17%) | 3 (75%)  1 (25%) | 43 (67%)  21 (33%) | 5 (29%)  12 (71%) | 0.004 |

For questions with multiple answer options the % indicates the percentage of answers within the transplant region. Percentages are rounded to the nearest whole number. Differences between groups were tested using a Kruskal-Wallis test for continuous variables and Chi-square test for categorical variables.

** Multiple answers could be given; therefore, percentage of respondents can exceed 100%. Differences are tested using Chi-square for each answer.

Abbreviations: KDIGO, Kidney Disease: Improving Global Outcomes; BTS, British Transplant Society

**Table S3: Kidney function assessment per transplant region**

|  | **Region** | | | |  |
| --- | --- | --- | --- | --- | --- |
| **Questionnaire item** | **Eurotransplant** | **Scandiatransplant** | **Southern Alliance** | **Other** | **P-value** |
| Initial GFR evaluation*, n* (%)  CKD-EPI  MDRD  EKFC  24-hour CrCl  Other | 15 (52%)  1 (3%)  0 (0%)  12 (41%)  1 (3%) | 2 (50%)  0 (0%)  0 (0%)  0 (0%)  2 (50%) | 45 (67%)  2 (3%)  1 (1%)  18 (27%)  1 (1%) | 8 (32%)  2 (8%)  0 (0%)  13 (52%)  2 (8%) | 0.002 |
| Use of cystatin C*, n* (%)  Creatinine-cysC combined  CysC alone  Only creatinine | 12 (41%)  3 (10%)  14 (48%) | 3 (75%)  0 (0%)  1 (25%) | 29 (43%)  1 (1%)  37 (55%) | 7 (28%)  0 (0%)  18 (72%) | 0.10 |
| Use of mGFR*, n* (%)  Always  Sometimes  Never | 11 (38%)  9 (31%)  9 (31%) | 4 (100%)  0 (0%)  0 (0%) | 38 (57%)  12 (18%)  17 (25%) | 7 (28%)  7 (28%)  11 (44%) | 0.06 |
| Use of mGFR tracer*, n* (%)*  Plasma iohexol  Urinary iohexol  Plasma 125I-iothalamate  Urinary 125I-iothalamate  Plasma 99mTC-DTPA  Urinary 99mTC-DTPA  Other/unspecified | 5 (25%)  1 (5%)  1 (5%)  1 (5%)  9 (45%)  0 (0%)  3 (15%) | 2 (50%)  0 (0%)  0 (0%)  0 (0%)  2 (50%)  0 (0%)  0 (0%) | 11 (22%)  2 (4%)  0 (0%)  1 (1%)  32 (64%)  3 (6%)  1 (1%) | 1 (7%)  0 (0%)  1 (7%)  1 (7%)  9 (64%)  2 (14%)  0 (0%) | 0.40 |
| Indexation of mGFR*, n* (%)*  No indexation  Indexed to BSA in mL/min/1.73m^2^  Both indexed and un-indexed | 5 (25%)  12 (60%)  3 (15%) | 0 (0%)  4 (100%)  0 (0%) | 11 (22%)  27 (55%)  11 (22%) | 0 (0%)  9 (75%)  3 (25%) | 0.34 |
| Lowest GFR threshold sufficient*, n* (%)  90 mL/min/1.73m^2^  80 mL/min/1.73m^2^  60 mL/min/1.73m^2^  Age-dependent | 1 (3%)  5 (17%)  1 (3%)  22 (76%) | 0 (0%)  0 (0%)  0 (0%)  4 (100%) | 2 (3%)  18 (27%)  3 (5%)  44 (66%) | 4 (16%)  10 (40%)  1 (4%)  10 (40%) | 0.13 |

For questions with multiple answer options the % indicates the percentage of answers within the transplant region. Percentages are rounded to the nearest whole number. Differences between groups were tested using a Kruskal-Wallis test for continuous variables and Chi-square test for categorical variables.

* Only asked in subgroup of respondents using measured GFR.

Abbreviations: GFR, Glomerular Filtration Rate; CKD-EPI, Chronic Kidney Disease Epidemiology; MDRD, Modified-Diet Renal Disease; EKFC, European Kidney Function Consortium; CrCl, Creatinine clearance; cysC, cystatin C; DTPA, diethylene-triamine-pentaacetate; BSA, Body Surface Area.

**Table S4: Donor risk assessment per transplant region**

|  | **Region** | | | |  |
| --- | --- | --- | --- | --- | --- |
| **Questionnaire item** | **Eurotransplant** | **Scandiatransplant** | **Southern Alliance** | **Other** | **P-value** |
| Lower age limit for donation, years | 18 [18;18] | 18 [18;20] | 18 [18;20] | 20 [18;21] | 0.16 |
| Higher age limit for donation*, n* (%)  65 years  70 years  75 years  80 years  80+ years  No age limit | 0 (0%)  1 (3%)  0 (0%)  2 (7%)  2 (7%)  24 (83%) | 0 (0%)  0 (0%)  0 (0%)  0 (0%)  0 (0%)  4 (100%) | 0 (0%)  9 (13%)  11 (16%)  8 (12%)  1 (1%)  38 (57%) | 2 (8%)  1 (4%)  5 (20%)  3 (12%)  1 (4%)  13 (52%) | 0.08 |
| Indications for OGTT*, n* (%)**  Elevated fasting glucose  Elevated HbA1c  Family history of diabetes  Presence of obesity  Presence of hypertension  Presence of dyslipidaemia  Presence of microalbuminuria  History of gestational diabetes  For all donors  Never | 23 (79%)  19 (66%)  12 (41%)  11 (38%)  4 (14%)  2 (7%)  4 (14%)  6 (21%)  4 (14%)  3 (10%) | 0 (0%)  0 (0%)  0 (0%)  0 (0%)  0 (0%)  0 (0%)  0 (0%)  1 (25%)  3 (75%)  0 (0%) | 39 (58%)  31 (46%)  22 (33%)  27 (40%)  2 (3%)  1 (1%)  12 (18%)  24 (36%)  19 (28%)  5 (8%) | 19 (76%)  15 (60%)  7 (28%)  13 (52%)  1 (4%)  0 (0%)  4 (16%)  6 (24%)  5 (20%)  1 (4%) | 0.005  0.046  0.36  0.25  0.18  0.33  0.79  0.43  0.045  0.77 |
| Measurement of blood pressure*, n* (%)**  Non-automated office measurement  Automated office measurement  24-hour ambulatory measurement  Multiple ambulatory measurements  Other/unspecified | 6 (21%)  14 (48%)  19 (66%)  2 (7%)  1 (3%) | 1 (25%)  1 (25%)  2 (50%)  1 (25%)  0 (0%) | 12 (18%)  34 (50%)  36 (54%)  21 (31%)  0 (0%) | 13 (52%)  9 (36%)  12 (48%)  5 (20%)  0 (0%) | 0.009  0.50  0.60  0.07  0.34 |
| Indications for 24-hour measurements*, n* (%)**  Office SBP >140 or DBP > 90 mmHg  Office SBP > 130 or DBP > 85 mmHg  Use of antihypertensive drugs  History of hypertension  In all donors  Never | 12 (41%)  5 (17%)  4 (14%)  2 (7%)  15 (52%)  0 (0%) | 2 (50%)  2 (50%)  2 (50%)  1 (25%)  1 (25%)  0 (0%) | 36 (54%)  18 (27%)  22 (33%)  16 (24%)  19 (28%)  3 (5%) | 13 (52%)  9 (36%)  8 (32%)  12 (48%)  8 (32%)  0 (0%) | 0.74  0.32  0.19  0.007  0.16  0.45 |
| Exclude donors based on hypertension if*, n* (%)**  Uncontrolled hypertension and/or end organ damage  Use of one antihypertensive drug  Use of two antihypertensive drugs  Use of three or more antihypertensive drugs  Well-controlled hypertension and other risk factors  Well-controlled hypertension and young age  Presence of microalbuminuria  Borderline hypertension | 23 (79%)  0 (0%)  4 (14%)  23 (79%)  7 (24%)  8 (28%)  18 (62%)  0 (0%) | 3 (75%)  0 (0%)  0 (0%)  2 (50%)  0 (0%)  3 (75%)  2 (50%)  0 (0%) | 64 (96%)  0 (0%)  14 (21%)  54 (81%)  12 (18%)  16 (24%)  54 (81%)  0 (0%) | 24 (96%)  0 (0%)  6 (24%)  18 (72%)  6 (24%)  10 (40%)  18 (72%)  0 (0%) | 0.03  N/A  0.57  0.46  0.63  0.10  0.18  N/A |
| ApoL1 in donors of African descent*, n* (%)  Yes, routinely  Yes, if donor is related to recipient  No | 4 (14%)  5 (17%)  20 (69%) | 0 (0%)  0 (0%)  4 (100%) | 12 (18%)  6 (9%)  49 (73%) | 3 (12%)  4 (16%)  18 (72%) | 0.73 |
| BMI cut-off*, n* (%)  No  ≤ 25 kg/m^2^  30 - 34 kg/m^2^  35 - 39 kg/m^2^  ≥ 40 kg/m^2^ | 10 (34%)  1 (3%)  9 (31%)  9 (31%)  0 (0%) | 0 (0%)  4 (100%)  0 (0%)  0 (0%)  0 (0%) | 14 (21%)  1 (1%)  25 (47%)  26 (49%)  1 (1%) | 3 (12%)  0 (0%)  10 (40%)  8 (32%)  4 (16%) | 0.15 |
| Offer weight loss intervention*, n* (%) | 15 (52%) | 1 (25%) | 57 (85%) | 20 (80%) | 0.001 |
| Type of weight loss intervention*, n* (%)***  Dietary intervention  Exercise intervention  Medication  Bariatric surgery | 13 (45%)  7 (24%)  7 (24%)  4 (14%) | 0 (0%)  1 (25%)  0 (0%)  0 (0%) | 52 (78%)  18 (27%)  15 (22%)  9 (14%) | 19 (76%)  8 (32%)  7 (28%)  1 (4%) | <0.001  0.93  0.66  0.50 |
| Decision making on ADPKD*, n*(%)**  Always exclude when positive family history  Use ultrasound for detection and exclusion  Use MRI for detection and exclusion  Use ultrasound/MRI based on age  Perform PKD gene mutation analysis  Exclude all young donors with positive family history  Never exclude when positive family history  Other/unspecified | 0 (0%)  14 (48%)  7 (24%)  14 (48%)  15 (52%)  6 (21%)  0 (0%)  0 (0%) | 0 (0%)  3 (75%)  0 (0%)  0 (0%)  1 (25%)  0 (0%)  0 (0%)  1 (25%) | 2 (3%)  26 (39%)  12 (18%)  23 (34%)  37 (55%)  9 (13%)  2 (3%)  4 (6%) | 2 (8%)  8 (32%)  9 (36%)  12 (48%)  14 (56%)  9 (36%)  1 (4%)  0 (0%) | 0.40  0.32  0.20  0.17  0.69  0.07  0.76  0.06 |
| Proteinuria test used for decision-making*, n* (%)  Only dipstick  24-hour urine protein excretion  24-hour urine albumin excretion  Spot urine protein/creatinine ratio  Spot urine albumin/creatinine ratio  All the above | 0 (0%)  3 (10%0  2 (7%)  4 (14%)  8 (28%)  12 (41%) | 0 (0%)  0 (0%)  0 (0%)  0 (0%)  4 (100%)  0 (0%) | 0 (0%)  15 (22%)  7 (10%)  7 (10%)  12 (18%)  26 (39%) | 1 (4%)  5 (20%)  4 (16%)  0 (0%)  6 (24%)  9 (36%) | 0.07 |
| Use of proteinuria for decision-making*, n* (%)  Use of albuminuria or both proteinuria/albuminuria  Only use proteinuria | 23 (79%)  6 (21%) | 4 (100%)  0 (0%) | 44 (66%)  23 (34%) | 13 (52%)  12 (48%) | 0.09 |
| Indications for donor kidney biopsies*, n* (%)  Donors with persistent isolated microscopic haematuria  Donors with proteinuria/albuminuria  Donors with haematuria and proteinuria/albuminuria  No routine biopsies | 3 (10%)  0 (0%)  3 (10%)  23 (79%) | 1 (25%)  0 (0%)  0 (0%)  3 (75%) | 18 (27%)  4 (6%)  6 (9%)  39 (58%) | 7 (28%)  4 (16%)  1 (4%)  13 (52%) | 0.24 |
| Exclude donors based on proteinuria if*, n* (%)**  Proteinuria > 150 mg/day (24-hour urine)  Proteinuria > 300 mg/day (24-hour urine)  Albuminuria > 100 mg/day (24-hour urine)  Albuminuria > 300 mg/day (24-hour urine)  Protein/creatinine ratio > 15 mg/mmol (spot urine)  Protein/creatinine ratio > 50 mg/mmol (spot urine)  Albumin/creatinine ratio > 3 mg/mmol (spot urine)  Albumin/creatinine ratio > 30 mg/mmol (spot urine)  Accept when kidney biopsy shows no abnormalities | 6 (21%)  15 (52%)  10 (35%)  8 (28%)  4 (14%)  10 (35%)  4 (14%)  13 (45%)  8 (28%) | 1 (25%)  0 (0%)  1 (25%)  0 (0%)  1 (25%)  0 (0%)  4 (100%)  1 (25%)  0 (0%) | 18 (27%)  32 (48%)  17 (25%)  16 (24%)  10 (15%)  14 (21%)  13 (19%)  23 (34%)  6 (9%) | 9 (36%)  11 (44%)  8 (32%)  2 (8%)  2 (8%)  1 (4%)  1 (4%)  12 (48%)  1 (4%) | 0.66  0.27  0.80  0.19  0.75  0.03  <0.001  0.53  0.03 |
| Decision-making in donors with haematuria*, n* (%)**  Exclude if persistent microscopic haematuria  Exclude when glomerular origin/proteinuria  Exclude when non-glomerular origin  Accept when normal biopsy and urological evaluation  Accept when normal biopsy  Accept when normal urological evaluation  Other/unspecified | 5 (17%)  13 (45%)  1 (3%)  14 (48%)  7 (24%)  4 (14%)  0 (0%) | 0 (0%)  2 (50%)  1 (25%)  1 (25%)  0 (0%)  1 (25%)  0 (0%) | 7 (10%)  27 (40%)  2 (3%)  42 (63%)  8 (12%)  8 (12%)  4 (6%) | 0 (0%)  10 (40%)  2 (8%)  14 (56%)  5 (20%)  6 (24%)  0 (0%) | 0.17  0.96  0.19  0.33  0.35  0.49  0.31 |
| Decision-making in donors with nephrolithiasis*, n* (%)  Routinely excluded, regardless of risk profile  Exclude if bilateral stones  Accept if low risk profile and no stones >4mm  Accept if low risk*, n*o stones >4mm and symptoms >5 yr  Accept if low risk*, n*o stones >4mm and symptoms >10 yr | 0 (0%)  8 (28%)  13 (45%)  6 (21%)  2 (7%) | 0 (0%)  1 (25%)  1 (25%)  1 (25%)  1 (25%) | 3 (5%)  17 (25%)  25 (37%)  14 (21%)  8 (12%) | 0 (0%)  10 (40%)  6 (24%)  7 (28%)  2 (8%) | 0.81 |
| Decision-making in donors using NSAIDs*, n* (%)  Accept if otherwise healthy  Accept if used for rheumatological disease  Accept when limited use  Demand stop based on age  Only accept when stopped  Depends on the NSAID | 2 (7%)  2 (7%)  3 (10%)  1 (3%)  19 (66%)  2 (7%) | 0 (0%)  0 (0%)  1 (25%)  1 (25%)  2 (50%)  0 (0%) | 13 (19%)  0 (0%)  4 (6%)  1 (1%)  44 (66%)  5 (8%) | 9 (6%)  0 (0%)  1 (4%)  1 (4%)  11 (44%)  3 (12%) | 0.049 |
| Decision making in cigarette smoking*, n* (%)  Routinely exclude smokers  Accept, but encourage cessation  Accept, but demand cessation <4 week before donation  Accept, but demand documentation of cessation | 4 (14%)  20 (69%)  4 (14%)  1 (3%) | 0 (0%)  2 (50%)  1 (25%)  1 (25%) | 0 (0%)  55 (82%)  11 (16%)  1 (1%) | 0 (0%)  20 (80%)  4 (16%)  1 (4%) | 0.01 |
| Use of donor risk tool by Grams et al NEJM 2016*, n* (%)  Routinely  For some donors  Use of different risk tool  No | 5 (17%)  10 (35%)  0 (0%)  14 (48%) | 1 (25%)  0 (0%)  0 (0%)  3 (75%) | 14 (21%)  15 (22%)  2 (3%)  36 (54%) | 7 (28%)  3 (12%)  1 (4%)  14 (56%) | 0.82 |
| Use of ESRD risk threshold*, n* (%)  1%  3%  5%  10%  Individualized threshold  No threshold | 6 (21%)  1 (3%)  0 (0%)  1 (3%)  16 (55%)  5 (17%) | 0 (0%)  0 (0%)  0 (0%)  0 (0%)  1 (25%)  3 (75%) | 7 (10%)  4 (6%)  3 (4%)  0 (0%)  41 (61%)  12 (18%) | 1 (4%)  1 (4%)  4 (16%)  0 (0%)  13 (52%)  6 (24%) | 0.11 |

For questions with multiple answer options the % indicates the percentage of answers within the transplant region. Percentages are rounded to the nearest whole number. Differences between groups were tested using a Kruskal-Wallis test for continuous variables and Chi-square test for categorical variables.

* Only asked in subgroup of respondents using measured GFR.

** Multiple answers could be given; therefore, percentage of respondents can exceed 100%. Differences are tested using Chi-square for each answer.

*** Multiple answers could be given; therefore, percentage of respondents can exceed 100%. Differences are tested using Chi-square for each answer. Answers were categorized by the researchers based on string answer variables.

Abbreviations: SBP, systolic blood pressure; DBP, diastolic blood pressure; ADPKD, autosomal dominant polycystic kidney disease; NSAID*, n*on-steroidial anti-inflammatory drug;

**Table S5: Donor follow-up practices per transplant region**

|  | **Region** | | | |  |
| --- | --- | --- | --- | --- | --- |
| **Questionnaire item** | **Eurotransplant** | **Scandiatransplant** | **Southern Alliance** | **Other** | **P-value** |
| Follow-up obligations*, n* (%)  Yes  No | 21 (72%)  8 (28%) | 3 (75%)  1 (25%) | 46 (69%)  21 (31%) | 7 (28%)  18 (72%) | 0.002 |
| Routine follow-up for donors*, n* (%)  Yes  No | 29 (100%)  0 (0%) | 4 (100%)  0 (0%) | 67 (100%)  0 (0%) | 24 (96%)  1 (4%) | 0.26 |
| Speciality ‘in charge’ of follow-up*, n* (%)  Nephrologist  Surgeon  General practitioner  General practitioner when good health, else nephrologist  Other/unspecified | 20 (69%)  2 (7%)  4 (14%)  1 (3%)  2 (7%)  0 (0%) | 4 (100%)  0 (0%)  0 (0%)  0 (0%)  0 (0%)  0 (0%) | 59 (88%)  4 (6%)  0 (0%)  1 (1%)  3 (4%)  0 (0%) | 18 (75%)  2 (8%)  0 (0%)  4 (17%)  0 (0%)  1 (4%) | 0.01 |
| Follow-up intensity*, n* (%)  Annually or more often  Every 2-4 years  Other/unspecified | 23 (79%)  4 (14%)  2 (7%) | 1 (25%)  3 (75%)  0 (0%) | 66 (99%)  0 (0%)  1 (1%) | 21 (88%)  3 (13%)  0 (0%) | <0.001 |
| Medical items part of routine follow-up*, n* (%)**  Blood pressure  24-hour urine proteinuria/albuminuria  Spot urine/dipstick  eGFR_creatinine_  eGFR_cystatin C_  24-hour creatinine clearance  Measured GFR  Lipid testing  Glucose/HbA1c testing  Body composition measurement  Medicine and vitamin review | 28 (97%)  7 (24%)  24 (83%)  27 (93%)  3 (10%)  2 (7%)  3 (10%)  19 (66%)  20 (69%)  17 (59%)  22 (76%) | 4 (100%)  1 (25%)  3 (75%)  4 (100%)  3 (75%)  0 (0%)  0 (0%)  3 (75%)  4 (100%)  3 (75%)  4 (100%) | 66 (99%)  28 (42%)  46 (69%)  63 (94%)  4 (6%)  16 (24%)  15 (22%)  57 (85%)  61 (91%)  49 (73%)  45 (67%) | 24 (96%)  7 (28%)  21 (84%)  23 (92%)  0 (0%)  1 (4%)  5 (20%)  18 (72%)  19 (76%)  15 (60%)  16 (64%) | 0.86  0.31  0.33  0.94  <0.001  0.04  0.41  0.17  0.03  0.43  0.42 |
| Routine psychosocial counselling in follow-up*, n* (%)  Yes  No  Unspecified | 1 (3%)  28 (97%)  0 (0%) | 3 (75%)  1 (25%)  0 (0%) | 18 (27%)  49 (73%)  0 (0%) | 3 (13%)  21 (88%)  1 (4%) | 0.002 |
| Out-of-pocket expenses for donor follow-up*, n* (%)  Yes  No  Unspecified | 3 (10%)  26 (90%)  0 (0%) | 0 (0%)  4 (100%)  0 (0%) | 2 (3%)  65 (97%)  0 (0%) | 5 (21%)  19 (79%)  1 (4%) | 0.04 |

For questions with multiple answer options the % indicates the percentage of answers within the transplant region. Percentages are rounded to the nearest whole number. Differences between groups were tested using a Kruskal-Wallis test for continuous variables and Chi-square test for categorical variables.

** Multiple answers could be given; therefore, percentage of respondents can exceed 100%. Differences are tested using Chi-square for each answer.
